# Capecitabine-Induced Leukoencephalopathy: A Case Report

**DOI:** 10.7759/cureus.85756

**Published:** 2025-06-11

**Authors:** Midhat Waheed, Ammara Yasmeen, Farzan Malik, Tanveer Fatima, Sheher Bano, Zubia Tarar, Harris Siddiqi, Noor Ul Ain

**Affiliations:** 1 Medical Oncology, Shaukat Khanum Memorial Cancer Hospital and Research Centre, Lahore, PAK; 2 Medical Oncology, Shaukat Khanum Memorial Cancer Hospital and Research Centre, lahore, PAK; 3 Endocrinology and Diabetes, Shaukat Khanum Memorial Cancer Hospital and Research Centre, Lahore, PAK; 4 Radiology, Shaukat Khanum Memorial Cancer Hospital and Research Centre, Lahore, PAK

**Keywords:** capecitabine, disease-free, drug withdrawal, leukoencephalopathy, neurological complications

## Abstract

Capecitabine, an antineoplastic agent, is an orally active fluorinated pyrimidine that is metabolized to the active form, fluorouracil (FU), by the enzyme thymidine phosphorylase. Neurologic complications secondary to capecitabine are uncommon, especially leukoencephalopathy, which develops shortly after starting treatment, is quite rare. This report details a young man who developed this complication after receiving just the first cycle, and discontinuation of the causative agent led to clinical and radiological improvement.

## Introduction

Capecitabine, an antimetabolite drug, is commonly used in various cancers including breast, colon, esophageal, gastric, neuroendocrine, and pancreatic cancers [1]. Side effects of diarrhea and palmar-plantar erythrodysesthesia syndrome are common with this drug, but neurological toxicities are seen in less than 10% of cases with leukoencephalopathy reportedly in hardly a few case reports in the past years [2]. 

Since the neurological complication is reversible if detected timely, clinicians should be aware of its presentation and management. 

Here, we report a case of toxic leukoencephalopathy caused by this drug, which was favored by the radiological findings, and timely discontinuation of the drug led to patient recovery.

## Case presentation

A 40-year-old male, a known diabetic with a history of toxic thyroid nodule, was treated in 2016 with radioactive iodine and is now battling moderately differentiated adenosquamous carcinoma of the second part of the duodenum. He underwent a Whipple procedure in March 2024 with histopathology suggestive of ypT3bN2. He was then planned for adjuvant chemotherapy with capecitabine and oxaliplatin for a total of eight cycles.

He was started on the first cycle of capecitabine at the dose of 1000 mg/m^2^ along with oxaliplatin (130 mg/m^2^) on April 24, 2024. Capecitabine was prescribed for 14 days. He remained well till 2/5/2024 when he presented to the emergency department with a complaint of motor aphasia lasting two hours, followed by slurring of speech, which eventually settled over the next 12 hours. On examination, power in all four limbs was intact, along with complete sensory examination, including all the cranial nerves. Cerebellar signs were negative. His vitals were in the normal range with blood pressure of 110/70 and heart rate of 89/min with sinus rhythm on ECG. The patient underwent CT brain with and without contrast on the same day, which was reported as unremarkable.

Blood workup including CBC, urea, electrolytes, calcium, magnesium, blood sugars, and phosphorus was unremarkable, which ruled out metabolic causes. Liver function test (LFT) was also performed, which turned out to be unremarkable (Table 1).

**Table 1:** Blood investigations

	Value	Reference range
White blood cell (WBC)	6.74 x 10^3^/µl	4.52-10.93
Hemoglobin (HB)	* 11.6 g/Dl	13.2-16.7
Platelet (PLT)	315 x 10^3^/µl	150-450
Sodium	136 mmol/L	136-145
Potassium	4.07 mmol/L	3.5-5.1
Creatinine	* 0.82 mg/dL	0.90-1.30
Bicarbonate	28.8 mmol/L	22-29
Ca, corrected	9.734 mg/dL	8.5-10.5
Magnesium	1.70 mg/dL	1.6-2.6
Phosphorus	4.3 mg/dL	2.9-4.7
Blood sugar level (BSL)	135 mg/dl	70-140

Initially, the impression was a transient ischemic attack, and he was advised to have an MRI brain for confirmation of diagnosis. Meanwhile, it was advised to hold all neoplastic agents till clinical recovery. 

**Figure 1:**
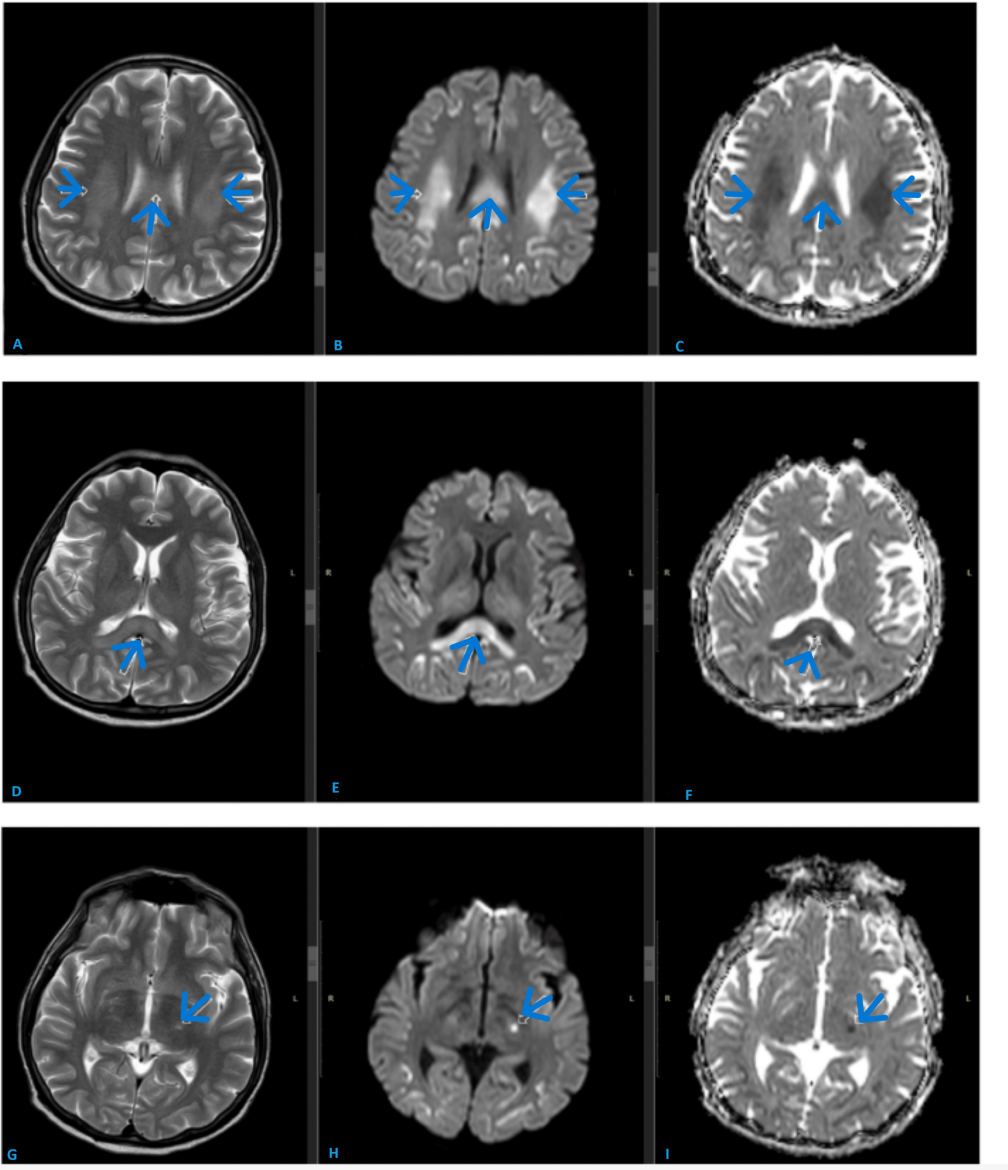
MR brain shows extensive abnormal T2 high signal with corresponding diffusion restriction in the bilateral frontoparietal white matter; trunk and splenium of corpus callosum and also a tiny focus in the left thalamus. Extensive abnormal T2 high signal is seen in the bilateral frontoparietal white matter(A);  trunk and splenium of corpus callosum (D) and also a tiny focus in the left thalamus (G). Corresponding diffusion restriction is also seen. Diffusion restriction is manifested as high signal on DWI sequences (B, E, and H) with low signal on ADC sequences (C, F, and I).

His MRI brain (Figure 1A) done four days later showed bilateral frontoparietal cortical and white matter signal abnormality with diffusion restriction. Findings were suggestive of capecitabine-induced leukoencephalopathy.

No metastatic disease was seen. By this time, all of his symptoms were resolved, and he had no focal neurological deficit. The case was discussed with neurology, and he had a CSF analysis done. The results are shown in Table 2. CSF cytology reported negative for malignant cells, and cultures were negative excluding meningitis/encephalitis and other inflammatory causes, which further favored the radiologic diagnosis.

**Table 2:** Cerebrospinal fluid (CSF) results.

	Value	Reference range
Albumin CSF	24.9 mg/dl	11-35
Protein CSF	35 mg/dL	15-45
Glucose CSF	* 81 mg/Dl	40-70
Lactate dehydrogenase (LDH) CSF	22 U/L	0-40
White blood cell (WBC) (/µL)	0.0 /µL	0
Red blood cell (RBC) (/µL)	0.0 /µL	0

**Figure 2:**
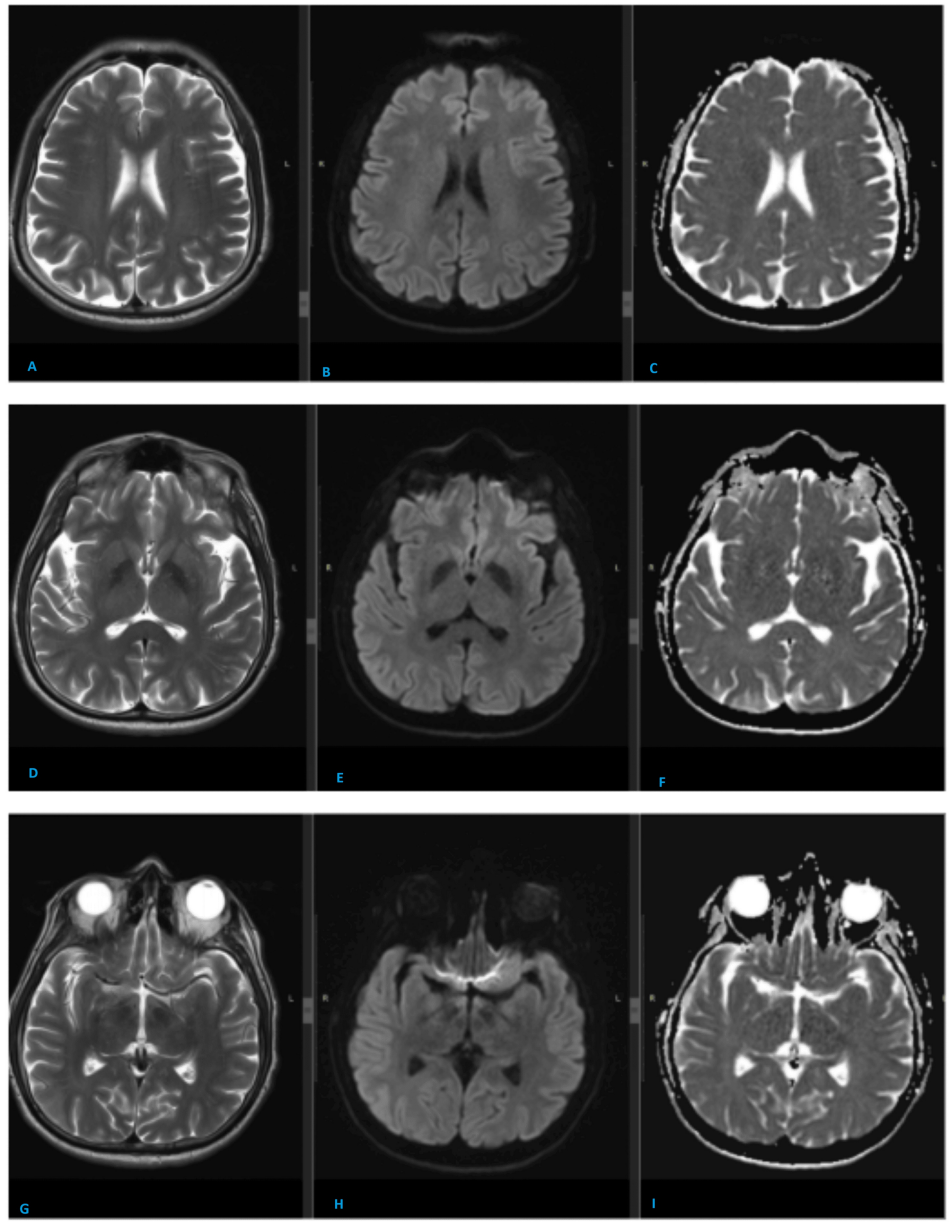
Follow-up MRI brain after approximately six weeks at same levels shows the interval resolution of abnormal signals and diffusion restriction. Follow-up MRI brain shows interval resolution of abnormal T2 high signals in the bilateral frontoparietal white matter (A); trunk and splenium of corpus callosum (D) and left thalamus (G). Also, there is interval resolution of diffusion restriction. Now, there is no abnormal signal on diffusion-weighted imaging (DWI) (B, E, and H) and apparent diffusion coefficient (ADC) sequences (C, F, and I).

His chemotherapy was held further as risks outweighed potential benefits and repeat MRI scan done (Figure 2B) after six weeks in June 2024 showed interval resolution of bilateral frontoparietal cortical and white matter signal abnormality and diffusion restriction. The patient is on surveillance since then, with no further neurological symptoms observed, and has remained disease-free for past one year.

## Discussion

Capecitabine-induced leukoencephalopathy was first described in 2004 [2]. The age of patients affected by this side effect ranges from 40 to 82 years. Capecitabine is converted in a three-step process to its active product, 5-fluorouracil (5-FU), an antineoplastic agent that mainly acts to inhibit thymidine synthesis and DNA replication. Although the exact mechanism of capecitabine neurotoxicity is not well understood, it is known that an intermediate metabolite of capecitabine, 5′-deoxy-5-fluorouridine (5′-DFUR), can cross the blood-brain barrier to enter the CSF and that thymidine phosphorylase, the last enzyme in the three-step cascade of capecitabine conversion to 5-FU, is found preferentially in the white matter tracts as compared to grey matter.

Risk factors associated with capecitabine-induced leukoencephalopathy include DPD deficiency (dihydropyridine dehydrogenase deficiency), renal Impairment, high dose or prolonged treatment, older age, hepatic dysfunction, previous CNS disease or brain metastases, and concomitant use of other neurotoxic drugs.

A separate study of 5-FU has also been shown to cause acute and delayed damage to the myelinated tracts of the central nervous system [3,4]. Radiologic findings include bilateral and symmetric lesions in the corpus callosum and corticospinal tract, showing hyperintensity on diffusion and FLAIR sequences with diffusion restriction presenting as acute or delayed central nervous system toxicity [5,6].

Oxaliplatin is a third-generation platinum compound used in several malignancies, especially related to the GI tract. This drug is known for its acute sensory toxicity with typical symptoms including throat discomfort, especially on swallowing cold items, sensitivity to touching cold items, muscle cramps and paresthesia, and dysesthesias of the hands, feet, and perioral region. These symptoms evolve over 24 to 96 hours and have the tendency to resolve in almost the same time frame. Another rare side effect observed with this drug is reversible posterior leukoencephalopathy syndrome (RPLS), which is confirmed by brain imaging [7]. The radiologic findings are usually suggestive of vasogenic edema in the subcortical white matter of the parietal and occipital lobes, with a dominant parieto-occipital pattern or watershed pattern.

These radiologic findings help in distinguishing neurotoxicity related to different chemotherapeutic agents. In our case, the patient symptoms and radiological findings favored more toward capecitabine-induced leukoencephalopathy , especially when the biochemical blood tests and CSF results turned out to be unremarkable. Toxic encephalopathy was diagnosed after other potential causes of neurological dysfunction were ruled out, and the patient's clinical symptoms fully resolved following the permanent cessation of chemotherapy, as was evident in repeat imaging in our case. 

## Conclusions

Clinicians using capecitabine should be aware of these rare side effects of the drug apart from the more common ones and should be watchful in patients who are at high risk of developing neurotoxicity. Patients experiencing new-onset neurological symptoms developing within days of starting treatment should be investigated by brain imaging after the complete metabolic workup. The radiological findings help in delineating the culprit drug and help in ruling out other differential diagnoses. 

Since the leukoencephalopathy can present with a wide range of symptoms, clinicians should have a low threshold for imaging for timely detection, as, fortunately, early withdrawal of the drug can lead to a complete resolution of symptoms.
